# Artificial intelligence-driven approach for patient-focused drug development

**DOI:** 10.3389/frai.2023.1237124

**Published:** 2023-10-12

**Authors:** Prathamesh Karmalkar, Harsha Gurulingappa, Erica Spies, Jennifer A. Flynn

**Affiliations:** ^1^Merck Data & AI Organization, Merck IT Centre, Merck Group, Bangalore, India; ^2^EMD Serono Research & Development Institute, Inc., Billerica, MA, United States

**Keywords:** patient-focused drug development, artificial intelligence, patient experience, text analytics, unmet needs, natural language processing, social media

## Abstract

Patients' increasing digital participation provides an opportunity to pursue patient-centric research and drug development by understanding their needs. Social media has proven to be one of the most useful data sources when it comes to understanding a company's potential audience to drive more targeted impact. Navigating through an ocean of information is a tedious task where techniques such as artificial intelligence and text analytics have proven effective in identifying relevant posts for healthcare business questions. Here, we present an enterprise-ready, scalable solution demonstrating the feasibility and utility of social media-based patient experience data for use in research and development through capturing and assessing patient experiences and expectations on disease, treatment options, and unmet needs while creating a playbook for roll-out to other indications and therapeutic areas.

## 1. Introduction

According to the US Food and Drug Administration, patient-focused drug development (PFDD) is a systematic approach to help ensure that patients' experiences, perspectives, needs, and priorities are captured and meaningfully incorporated into drug development and evaluation (US Food Drug Administration., 2022). The US Food and Drug Administration's guidelines serve as a boost for healthcare organizations to consider the use of social media to identify what is important for patients. Increasing digital participation on social media sources provides an opportunity to pursue patient-centric research and drug development by understanding the experiences and needs of patients. Drug development efforts benefit from including patient and caregiver social media listening (SML) as a component of a PFDD strategy. Identifying relevant data points from social media posts has been one of the major focus areas for researchers before applying methodologies to extract insights. Identifying patient and caregiver posts and/or tweets will help subject matter experts (SMEs) focus on experiences shared by individuals to synthesize optimal strategies for asset and medication research. This leaves organizations with potentially significant numbers of untapped data points, which could provide valuable insights prior to designing research and development pipelines.

There have been several studies published in this area where experts have leveraged social media data for training machine learning (ML) or natural language processing (NLP) to develop point solutions. Koss et al. proposed several use cases explaining theoretically the fundamentals of using social media in drug development scenarios (Koss et al., 2021). Furthermore, the authors explain use cases, such as identification and prioritization of unmet patient needs to ensure that patient perspectives are represented, characterization of the target population to develop a target profile of a drug, recruitment of patients during clinical trials, and detection of adverse events of drugs. Schmidt et al. explored the use of quantitative SML to PFDD (Schmidt et al., 2022). The authors describe the potential strengths and weaknesses of quantitative SML approaches coupled with computational techniques to build drug discovery or development pipelines. However, the authors do not cover specifics about experiments and results obtained using such methods. Fang et al. discussed the use of qualitative data generated from interviews with cancer patients in a controlled manner to characterize the use of NLP for extracting insights (Fang et al., 2022). The authors further discuss the use of multiclass classifiers to predict labels, such as symptom and quality-of-life impact among others, based on encoder representations and similar techniques. Based on the observations from this article, it seems that the authors could not conclude on the quality/accuracy of the model due to the insufficient size of the sample. Khanbhai et al. (2021) performed a systematic review of multiple ML and NLP techniques in a controlled environment to explore performance measures on social media data extracted from free-text comments held within structured surveys. Le Glaz et al. (2021) proposed a methodology using social media data and literature from public sources to identify and extract symptom severity and therapy effectiveness based on a random sample process. This approach documented the potential advantages and pitfalls of using certain techniques on social media and literature data. Rozenblum and Bates (2013) and Rozenblum et al. (2017) summarized several studies and techniques to understand patient experiences and engagement strategies from social media data. The research outlined above showcases potential methodologies that could be well suited for conducting efficient patient engagement strategies from social media data for a specific task or activity. There were other techniques evaluated on social media data in the context of patient experiences and engagements; however, there is limited literature that explains enterprise-level, customizable, solutions that could be extendable to new therapeutic areas based on previously trained models. In subsequent sections, we outlined a solution and its potential benefits when expanding the learnings and creating a step-by-step plan to expand this solution to other indications or therapeutic areas.

Here, we propose and demonstrate a holistic social media-based scalable artificial intelligence (AI) and NLP solution, assessing patient experiences and expectations on disease, treatment options, and unmet needs.

## 2. Methods

### 2.1. High-level design overview

**Figure 1:**
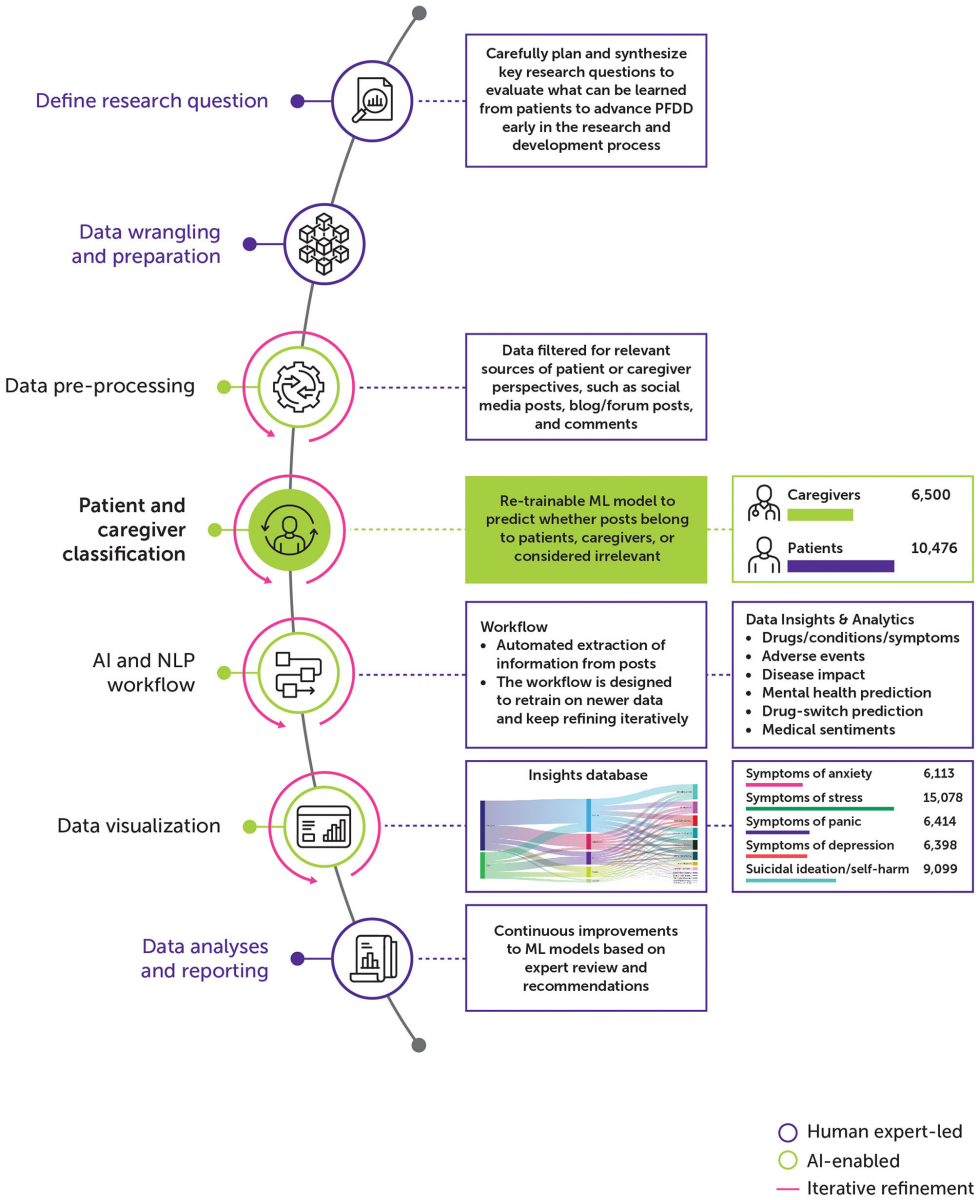
Overview of AI-enabled solution.

The solution has two major phases: (1) expert-driven finetuning and (2) AI-enabled decision-making to identify valuable patient and caregiver insights from the data (Figure 1). The first step is to carefully define, plan, and design key research questions that should be considered when building a potential asset research and development pipeline for a specific indication or set of indications with a therapeutic area of interest. Some of the research question examples are: What do patients think about their current treatment? How do patients experience unmet needs? What do patients want in a treatment for the condition? The questions were crafted in correlation with patient experiences and expectations.

The next step is to establish a data extraction pipeline to fetch data from relevant social media sources based on pre-defined keywords and terms. Keywords and terms can be identified based on indications/therapeutic areas of interest. Third, human analysts can identify and select data sources for inclusion or exclusion from the dataset to process. Team member-defined keyword inclusion criteria for indications were then refined, as data were reviewed for inclusion into the data corpus for the AI solution to leverage. Fourth, using custom trained machine learning model, data can be filtered and aggregated to label as “patient,” “caregiver,” or “irrelevant.” Fifth, algorithmic coding of study concepts of interest, as guided by the research questions, can be conducted for patient and caregiver-specific data. Subsequently, a set of NLP techniques can be applied for automated extraction of information indicative of patient experiences, such as drugs/conditions/symptoms, adverse events, disease impact, psychosocial health prediction, drug-switch prediction, and medical sentiments. The generated insights can be leveraged by the end users in the form of interactive analytical views. Details of specific components from AI-enabled phases of the solution will be explained in subsequent sections. The initial benefit of this approach is the ease of adoption and extending the same to new indications of interest from different therapeutic areas. The solution is designed to handle integration of new indications or therapy areas as per future drug development pipeline strategy with minimal human intervention. Further, the solution is also equipped to ingest newer data sources to address patient unmet needs. This approach is scalable and enterprise-wide to build a Patient Experience Knowledge Hub for all new assets in the research pipeline.

### 2.2. Dataset setup

Approximately 150,000 posts and data points (before applying any preprocessing or filtering) across 2 key indications of interest in the area of Head & Neck and Esophageal cancer were used to train, customize, and build the models as well as perform large-scale inferencing. An enterprise, licensed SML platform was used to collect data from various online health-specific communities in English from 7 countries from January 2020 to April 2022. Data collection was based on SME-defined queries, and NLP techniques coupled with ML algorithms were applied for data cleansing. The data were mixed for different disease sub-types of Head & Neck and Esophageal cancer to add variations in the model training. The data were curated from Twitter^®^ updates, Twitter^®^ replies, forums, blog posts, Twitter^®^ mentions, print news, review types, blog comments, Reddit^®^ posts, WordPress^®^ posts, Quora^®^ answers, WordPress^®^ comments, Quora^®^ comments, podcast messages, and classified messages. Supplementary Table S3 shows a sample of the search query used for extracting an initial set of data from the SML platform.

Based on the analysis of the raw data with human experts, our approach uses a five-step pre-processing technique to prepare for downstream AI solution.

- Filter out irrelevant sources from the raw dataset which removes posts from News, Podcasts, Video comments, etc.;
- Continuous refinement to search query based on the addition/updating of keywords to ensure quality data for key business objectives;
- Removal of personally identifiable information (PII) to protect the privacy of the users including any names, addresses, and postal codes;
- Removal of specific sub-sources irrelevant to AI solution including but not limited to YahooFinance^®^ and Daily Political^®^
- Removal of mentions of user profiles from tweets to avoid processing any further personal data.

### 2.3. AI-enabled solution

#### 2.3.1. Classifying patient and caregiver posts based on negative learning

Due to the nature of the social media data and the abundance of spam or irrelevant posts coming in as a result of a search query on multiple sources, it is crucial to filter the data from the dataset to ensure that the AI and NLP models are trained on qualitative data rather than just a significant quantity. To ensure that the training of the model is contextual to business questions shared by experts, a clear definition of “patient,” “caregiver,” or “irrelevant” is vital to ensure that no gaps are observed.

The traditional approach to training a model for classification tasks is called positive learning, which leverages data and corresponding labels. If the given label is false, the model will likely overfit the faulty information. In contrast, negative learning leverages the information from complementary labels to train the model. The complementary labels are the labels of all classes, except the class of the given label (Kim et al., 2019). By training the model not to select one of the complementary labels, it is possible to prevent the model from overfitting to noisy labels. The mathematical explanation is as follows:

Suppose, we have n labels {1, …, n} where positive labels are represented as PL and negative labels are represented as NL, and one of the labels y is given to a sample x as the “supposed to be true” label. The posterior probability of providing true information using positive learning is P(True|PL), which is the same as P(y|x). The posterior probability of providing true information by negative learning using randomly selecting a complementary label $\bar{y}$ from {1, …, n}/{y} is as follows:


$$\begin{array}{l} P\left(True|NL\right)=P\left(y|x\right)+\;\frac{1}{n-1}\;P\left(y_{0}|x\right)+\ldots +\frac{1}{n-1}\;P\left(y_{n-1}|x\right)\\ \;=\;P\left(True|PL\right)+\frac{1}{n-1}\;P\left(y_{0}|x\right)+\ldots \\ \;+\frac{1}{n-1}\;P\left(y_{n-1}|x\right)\geq P\left(True|PL\right) \end{array}$$

Even in the worst scenario, where we are 100% sure that y is the true label, we have P(y|x) = 1, which means P(True|PL) is also 1 and P(y_0_|x), …, P(y_n − 1_|x) are all 0, then P(True|NL) is equal to P(True|PL). However, we can prove that negative learning will never be inferior to positive learning in providing true information. To implement negative learning, the key is the loss function. With positive learning, the model should maximize the probability of the given label using the cross-entropy loss function:


L(f,y)=−∑k=1cyk log pk

With negative learning, the model should minimize the probability of the complementary labels using the loss function, where ${\bar{y}}_{k}$ is the complementary label that is randomly selected from all labels except the given label (Kim et al., 2019):


$$L\left(f,\bar{y}\right)=-\sum_{k=1}^{c}{\bar{y}}_{k}\;\log(1-p_{k})$$

#### 2.3.2. Medical sentiment classifier

Understanding the efficacy and performance of the treatment is one of the crucial aspects of the success or failure of a product or asset, in conjunction with how patients and caregivers describe experiencing treatment. Standard ML or NLP approaches have certain limitations, where they either have fixed labels in terms of “positive” and “negative” or they require significant labeled data to train custom classifiers. Traditional sentiment classifier categorizes input text into three categories: “positive,” “negative,” or “neutral.” Our solution proposes a medical sentiment classifier using a zero-shot classification methodology. Based on the efficacy of drug treatment, the medical sentiment classifier categorizes input text into three categories. After medical treatment, these classes are patient health is getting better, deteriorating, or the medical condition still exists. Manually labeling the dataset of treatment experiences in relation to patient health requires time and effort from domain experts. Hence, there is a strong case to use methods such as zero-shot classification. Our approach/study implements and evaluates zero-shot classification using a pre-trained denoising autoencoder from the transformer (BART) model (Lewis et al., 2020).

An entailment approach was used as a relation identification task to identify relations between two pieces of input texts where one contains subject and object for candidate relation while the other contains the description of the relation. The model returns a binary response as output, indicating whether the meaning of the description explains the relation between the subject and object (Obamuyide and Vlachos, 2018). Zero-shot learning may include unseen labels during classification. Additionally, the number of labels can be changed dynamically for each problem. In traditional text classification, a model is trained on already observed labels, and labels are not considered for any decision process. Class labels are mere numbers, while traditional classifiers try to fit the best decision boundary on the basis of a given trained dataset. The entailment approach suggests that each text to be classified can be posed as a premise. The labels themselves can be considered for text classification. Implementation of zero-shot text classification is outlined as follows:

1. Each input text is considered as a premise of the natural language inferencing (NLI) task.
2. Let us assume for given text input x, there are n classes <y_1_, y_2_, y_3, …_, y_n_>. For zero-shot classification, this number can vary for different inputs.
3. Each class label is converted into a hypothesis. This can be performed by generating sentences that semantically capture the aspect of a given class. For instance, the class label “*treatment*” can be converted into a hypothesis by appending a sentence like “*The text is about _____*.”
4. In this case, the hypothesis becomes “*The text is about treatment*.” This should be performed for all class labels associated with the given input.
5. Once all class labels are converted into hypothesis, text classification can be posed as an NLI task. Each pair of premise hypotheses is fed to a model that is fine-tuned on the NLI task.
6. The entailment scores for each label are calculated as the output of the NLI-trained model.
7. Furthermore, softmax is applied to the entailment scores of each class label. For n classes, there are n corresponding entailment scores.
8. The class label with the maximum probability score is assigned as the predicted class.

Our solution proposes a methodology that can be used to extract high-quality classified data points using zero-shot classification. To avoid additional training cost, a BART model fine-tuned on the NLI task was chosen. Initially, a set of three labels was created that represented classes of medical sentiment classifiers. However, it was observed that slight changes in these hypotheses can generate different results. To overcome this bottleneck, two different sets of hypotheses were used. The second set of hypotheses consists of similar sentences except for additional context added to the first set. Hypothesis Set 1 used the following: (a) health is deteriorating after treatment; (b) medical condition is still present; or (c) health is improving due to treatment; Hypothesis Set 2 used the following: (a) medical condition is deteriorating; (b) medical condition still exists; or (c) medical condition has subsided. Modifying these hypotheses slightly leads to variation in zero-shot classification output. The implementation using two different sets of hypotheses is outlined below:

1. For the given input text, a score of entailments is computed for the three hypotheses in Set 1. Softmax on each entailment is applied to get the final probability score for the predicted label.
2. The above step is repeated for Hypothesis Set 2.
3. Those inputs are selected that have the same output prediction and the probability scores are average for both sets of hypotheses.

One of the goals of this study is to implement and evaluate how general corpus trained the BART model can be used for medical sentiment classification. As no true labels were present and there should be minimum involvement of human experts, a suitable threshold was found to select high-quality data for further refinements and improvements.

#### 2.3.3. Psychosocial health prediction

According to recent studies, the detection and analysis of psychosocial health can be performed based on standard symptoms or signs associated with the pre-defined labels. Mental illnesses or psychosocial health disorders may be caused by a range of conditions that affect mood, thinking, and behavior and can result from stressful life situations, chronic medical conditions, traumatic experiences, or previous mental illness^1^. Psychosocial health disorders have been observed as a result of the diagnosis of a life-threatening disease or the impact of treatments on day-to-day functioning (Happell et al., 2020). Psychosocial health disorders can be labeled as “stress,” “depression,” “anxiety,” “panic,” or “suicidal.”

The objective of our approach is to identify patients or caregivers referring to cases of psychosocial health conditions observed as a result of the impact caused by the cancer or the treatment effects on day-to-day activities. For example, patients expressing anxiety due to shaking of hands or legs make them unable to perform common activities such as dropping kids at school, enjoying the food, etc.

Here, we describe an approach that investigated a cardiffnlp/Twitter-roBERTa-base (Barbieri et al., 2020) supervised ML approach to predict and label posts from patients and caregivers to understand if they discuss psychosocial health disorders, such as “stress,” “depression,” “anxiety,” “panic,” or “suicidal,” caused by the diagnosis of a life-threatening disease. Our approach starts with building initial definitions and understanding of how the psychosocial health disorder in our case should be reviewed or used for predictions. A sample of such definitions that were created as an initial skeleton for disorders is given in Table 1. The definitions and descriptions for the psychosocial health labels are created based on consultation and guidance from experts within our organization in the area of PFDD to ensure that the data are classified correctly.

**Table 1:** Sample definitions for training initial ML model (Lovibond and Lovibond, 1995).

**Depression**	**Anxiety**	**Stress**
Not being able to experience positive feelings	Being aware of mouth dryness	Getting upset at trivial things
Could not seem to get going	Experience breathing difficulties (breathlessness, excessive rapid breathing in the absence of physical exertion)	Over-reacting to situations
Feeling like you have nothing to look forward to	Feeling of shakiness	Finding it difficult to relax
Feeling lack of self-worth	Relief when feelings that cause anxiousness end	Getting upset rather easily
Feeling life is not worthwhile	Feelings of faintness	Using a lot of nervous energy
Not being able to find enjoyment	Sweating/perspiring in the absence of high temperature or physical exertion	Getting impatient when delayed in any way in simple chores
Feeling downhearted and blue	Feeling worried about situations in which one could panic and make a fool of myself	Feeling in a state of nervous tension
Unable to become enthusiastic about anything	Experience of trembling	Being intolerant of anything that keeps one from getting on with tasks at hand
Feeling that life is meaningless		Getting agitated
Finding it difficult to work up the initiative to do things		

#### 2.3.4. Symptom severity

There have been several advancements in terms of managing, preventing, and diagnosing symptoms associated with cancer or similar indications; however, patients still endure a significant experience, regardless of the stage of the disease they are in or the treatment that they are undergoing (Omran and McMillan, 2018). It is important to understand what type of symptoms are found to be more troublesome for patients or caregivers in terms of managing or handling physical, social, emotional, or cognitive behaviors. Patients have experienced differing levels of discomfort due to symptoms as a result of being diagnosed or treated at different stages of the indication. Identification of the severity or the burden of symptoms or set of symptoms can assist in effectively reducing the damage caused to the patients. Some of the examples of the symptoms reported by patients or caregivers that are used as reference/taxonomy to predict or compute their corresponding severity based on the contextual information available are: swelling that does not heal, a sore that does not heal, red patch in the mouth, white patch in the mouth, lump in the head area with pain, foul mouth odor not explained by hygiene, hoarseness in voice, change in voice, nasal obstruction, persistent nasal congestion, unusual nasal discharge, loosening of teeth, dentures that no longer fit, unexplained weight loss, fatigue, ear infection, persistent cough, coughing up blood, feeling there is something stuck in the throat, and numbness in the mouth that will not subside.

**Figure 2:**
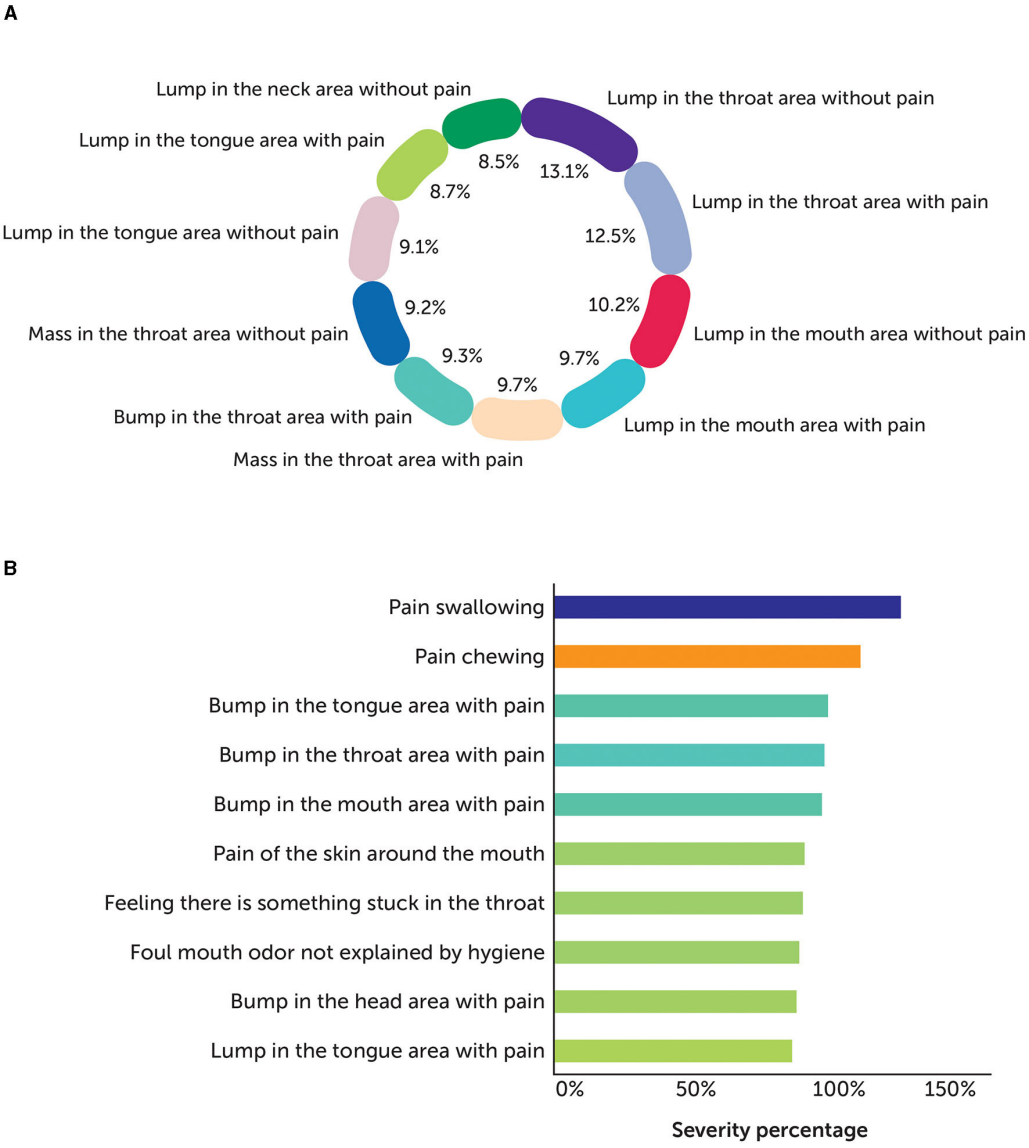
Sample view of **(A)** symptom distribution and **(B)** top 10 symptoms by severity.

To effectively identify and assess the severity, we developed an approach by combining a series of different techniques. The fundamental technique is “aspect-based sentiments,” which computes the level of tonalities at an attribute level either identified from texts or explicitly defined by domain SMEs. Here, we experimented with the Twitter-roBERTa-base for the sentiment analysis model (Barbieri et al., 2020) to identify sentiments, keeping symptoms as aspects for the predictions. Due to the nature of the social media data, it is observed that it is necessary to further boost or negate the scores based on additional factors, such as (1) degree of the words—comparative vs. superlative; (2) explicit use of negations to emphasize on the negative tonality; (3) use of slang or urban dictionaries, especially used on social media; (4) use of emoticons to further stress on negations. An example of how the results from symptom severity are consumed and used by domain SMEs to understand what patients or caregivers discuss in their posts is given in Figure 2.

#### 2.3.5. Potential side effects prediction

To effectively understand the experience and expectations of patients, it is good practice to identify potential side effects from textual data. This can assist in understanding what patients go through during the diagnosis and treatment of cancer or other diseases. Based on certain studies and estimations, it is observed that 90% of the potential side effects of assets are either not reported or missed due to the nature of the data (Sukkar, 2015). Social media is considered to be one of the untapped sources of information to effectively identify potential side effects from textual data (Burkhardt et al., 2020). Identification of potential side effects is one of the crucial steps in identifying patient experience as it can help SMEs understand the safety of the approved assets, the efficacy of the asset, and how companies can avoid such side effects during asset development. As per the literature and studies performed, it is suggested to validate significant data points by experts to ensure that the accurate asset–side effect pair is extracted from the texts (Huang et al., 2022).

There are several techniques that have been experimented with and evaluated based on the concepts of machine learning or deep learning. However, it is observed that many of the techniques need significant data and labeling due to variations in the way a term is used. For example, “puffy face,” “puffiness and redness on face,” and “blown up face” are some cases where most of the time a specific side effect is mentioned in layman's language. Hence, our solution uses a unique approach with a combination of a BERT-based token classifier along with a custom-trained transformer classifier model (John Snow Labs., 2021) on texts mentioned and expressed on one of the known sources called “Twitter.”

## 3. Experimental setup and outcomes

### 3.1. Negative learning—Results and observations

#### 3.1.1. Experimental settings

We manually annotated approximately 2,950 random records from Blog, Tweet, and Forum datasets, out of which 90% was used for training and the remaining 10% used for testing as the clean dataset with three labels, namely, “patient,” “caregiver,” and “irrelevant” to conduct our experiments. The source distribution is shown in Table 2.

**Table 2:** Distribution of labeled data by sources for patient and caregiver classification.

**Sr. No**.	**Data source**	**Indication type**	**Counts**
1	Forums	Head and neck	394
2	Blog post	Head and neck	356
3	Twitter	Head and neck	775
4	Forums	Esophageal	358
5	Blog post	Esophageal	346
6	Twitter	Esophageal	739

To test the performance of the model in denoising and self-supervising, we added random noise to the dataset. To generate noise, we randomly selected α (the noise ratio) percent of records, and for each of them, we randomly selected all possible labels, except for the given one as the noisy label.

To classify data, we utilized a RoBERTa-base model (Barbieri et al., 2020) trained on approximately 58 million tweets. The input sequence was truncated to 512 tokens. We used a batch size of 16 and an Adam optimizer with a learning rate of 1e^−5^. Epochs were set to 20.

#### 3.1.2. Complementary labels

We considered two options for generating complementary labels as follows: whether the complementary labels are fixed or changed after each epoch. If the complementary labels are fixed, the model learns the same negative labels after each epoch. After training, the model learns the negative labels that the samples should not belong to. As a result, the model will have much lower confidence in the noisy labels than the clean labels. By separating the clean and noisy data by confidence, we can train the model with clean data and then apply the trained model to noisy data to denoise them.

**Figure 3:**
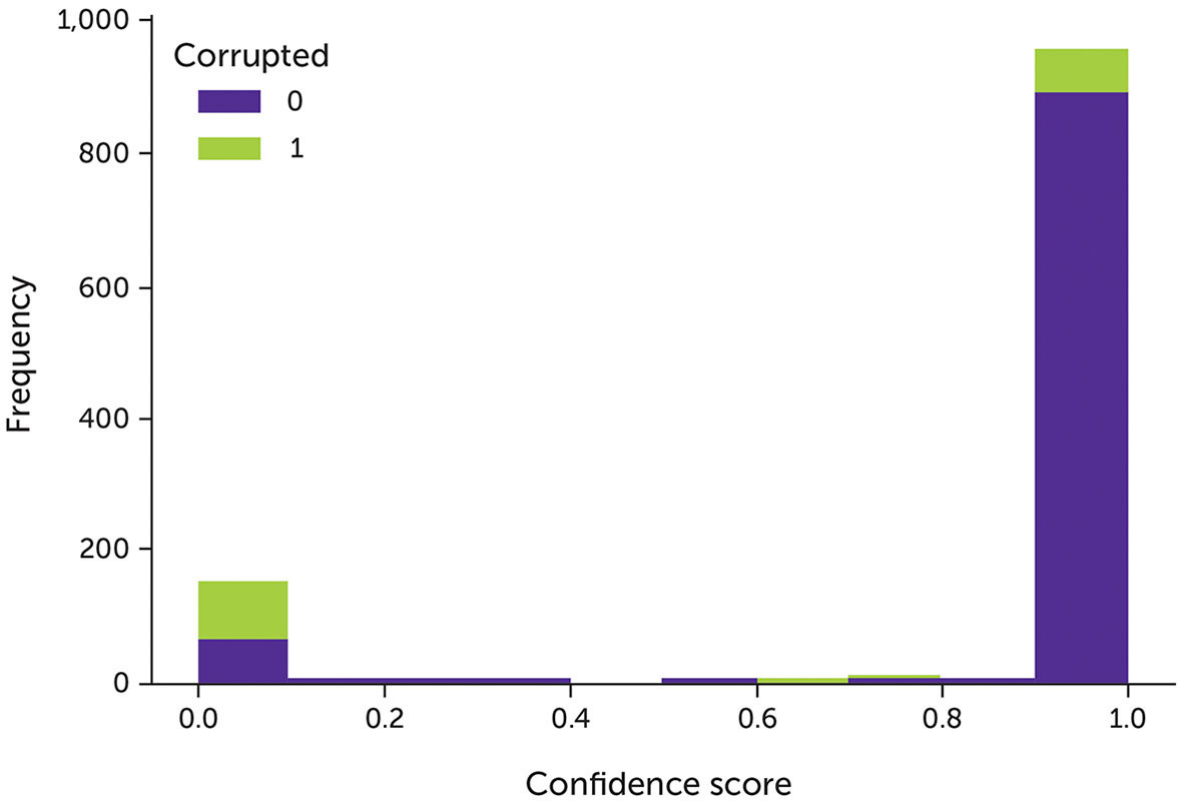
The model's confidence of the data with 30% noise after trained with fixed complementary labels. Green indicates the corrupted data; purple indicates the original clean data.

The majority of noisy data were assigned with very low confidence, whereas most of the clean data were assigned with very high confidence (Figure 3). If the complementary labels are changed, the model can learn new negative labels after each epoch. However, after a certain number of epochs, the model can learn all the possible complementary labels, which is the same as training the model with all the given labels. To solve this issue, we proposed a new approach to train the model: if the model can denoise the data, it should predict different labels for noisy data. During the training process, we tuned the hyperparameters based on the minimum difference between the accuracy of the model and the inherent accuracy of the data, which were inferred by labeling an acceptable number of random samples manually.

**Figure 4:**
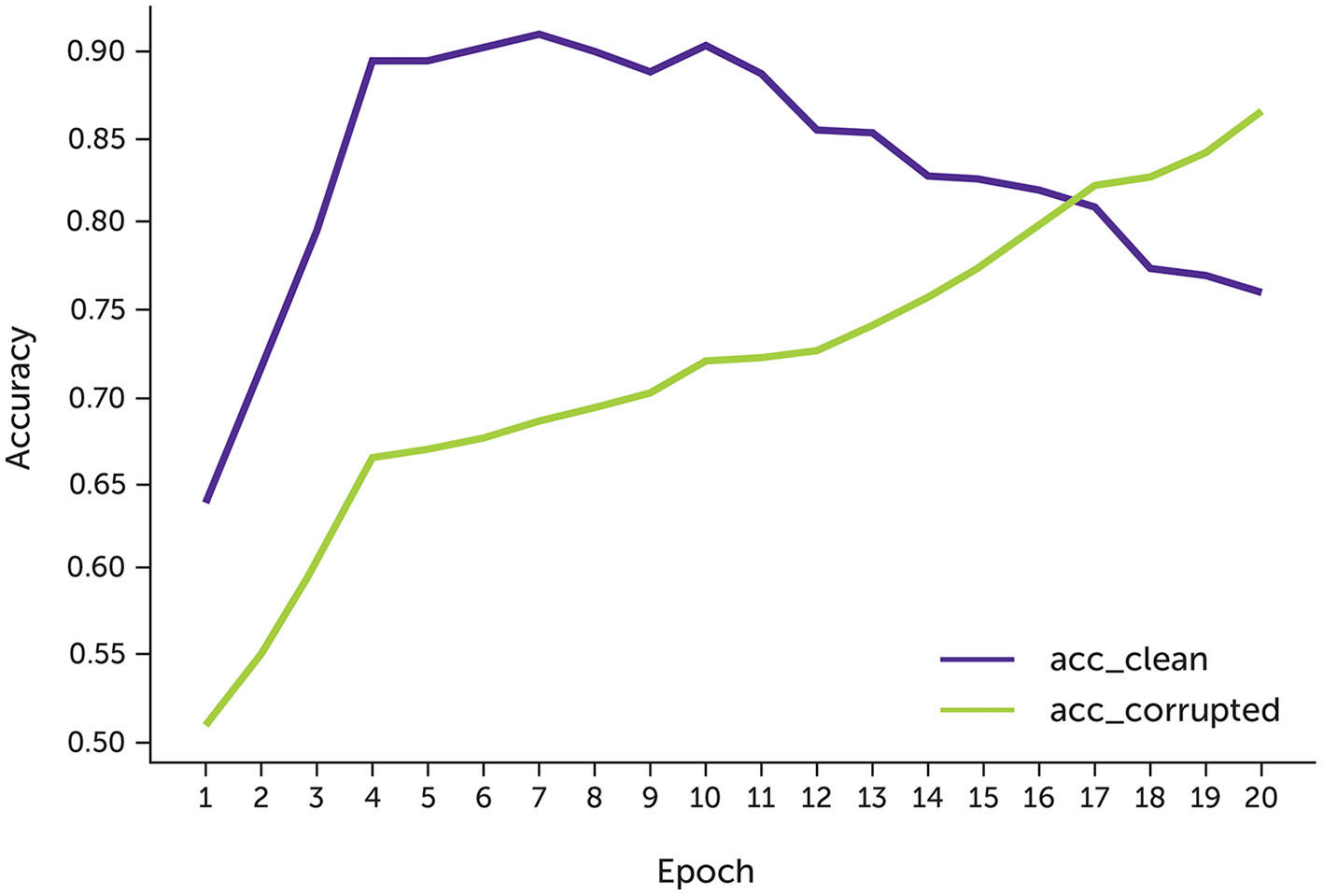
The accuracy of the data with 30% noise.

The model had higher accuracy on the clean data approximately the 9th epoch since the model's accuracy on the noisy data is close to 70% (Figure 4). After that, the accuracy of the clean data drops to 70% since most possible complementary labels have been learned.

We experimented with two types of complementary labels: fixed and changed complementary labels. With fixed complementary labels, we randomly selected a complementary label for each sample once before training. With changed complementary labels, each sample was randomly assigned a complementary label after each epoch.

#### 3.1.3. Fixed complementary labels

With fixed complementary labels, we follow a 3-stage pipeline proposed by Kim et al. (2019), except the complementary labels remain the same after each epoch. The results from fixed complementary labels are shown in Table 3.

**Table 3:** Fixed complementary label results.

**α (noise %)**	**20**	**30**	**40**	**50**	**60**	**70**
Accuracy (%)	87.47	89.12	80.47	67.81	44.78	11.32
Macro F1 (%)	80.58	86.26	77.16	56.64	39.62	11.50

#### 3.1.4. Changed complementary labels

The results from changed complementary labels are shown in Table 4. The overall performance of changed complementary labels was superior vs. fixed complementary labels. This could be due to the limitations in tuning the hyperparameters of fixed complementary labels since the information of the whole dataset was needed to denoise itself. The common approach of splitting a part of the dataset into a validation set was not possible. As the next step, we plan to experiment with improving negative learning based on advanced loss function and adding different labels to analyze the performance of the model (Kim et al., 2021). The immediate next steps include incorporating learning from this research into additional therapeutic areas and data sources.

**Table 4:** Changed complementary label results.

**α (noise %)**	**20**	**30**	**40**	**50**	**60**	**70**
Accuracy (%)	92.30	88.74	87.34	78.44	54.01	18.63
Macro F1 (%)	90.14	85.67	82.18	72.01	46.47	18.16

### 3.2. Medical sentiment

To extract high-quality predictions, we found a suitable confidence threshold. The goal was to extract medical sentiments without labeled data. An independent test with distribution as mentioned in Table 5 was used to evaluate the model performance. After manual evaluation, a threshold of 90% confidence score was chosen on 500 sample data points from Blogs, Tweets, Reviews, and Forums. The confidence score is used as a threshold to determine the result cutoff for the BART model. Classified posts above this threshold were evaluated by two human experts. A total of 200 posts from Twitter were randomly sampled from this set to ensure that there was no bias toward high-confidence score tweets in this threshold range. These 200 posts were checked for the given prediction, and accuracy was used to measure the performance of the model. Each prediction was assigned to a correct or incorrect label. Table 6 includes representative sample posts from this manual review. Additional examples are presented in Supplementary Table S1.

**Table 5:** Distribution of independent test set by sources from social media across all algorithms.

**Sr. No**.	**Data source**	**Indication type**	**Counts**
1	Forums	Head and neck	2,003
2	Blog Post	Head and neck	2,886
3	Twitter	Head and neck	5,900
4	Reviews	Head and neck	500
5	Forums	Esophageal	462
6	Blog Post	Esophageal	1,591
7	Twitter	Esophageal	2,000
8	Reviews	Esophageal	200

**Table 6:** Sample output of medical sentiment classification.

**Posts**	**Label**
I was diagnosed with throat cancer in November 2020 and had my larynx removed in December. I am 9 months down this journey and doing well. I had 6 weeks of radiotherapy and 5 chemo treatments. Would be good to know if any one else has had the same type of cancer and how they feel.	Recovery
I thought this was touching, too. Prayers for him and his family. My husband's sister died from a new drug to help her cancer. If she didn't take the drug, she would have to drain her lungs on a daily basis. She opted for the drug. The side effects of the drug were horrible compared to her health issue. One of the side effects of the drug was throat cancer. She developed a huge tumor in her throat and wasn't able to eat or swallow. It was this side effect that killed her.	Deteriorating

To evaluate the performance of the model, a prediction was considered to be correct if it was agreed upon by two human evaluators. Out of 200 predictions, 165 were marked as correct. For 200 randomly sampled tweets, the zero-shot classifier had an accuracy of 83%.

### 3.3. Psychosocial health

A test set of approximately 2,000 data points from Blogs, Tweets, Reviews, and Forums were created for human evaluation. A similar approach was employed to evaluate the identified and tagged psychosocial health disorders associated with the posts, which were then manually analyzed by human experts to further refine and fine-tune the model. After four iterations of review and refinements by SMEs, the definitions of psychosocial health disorders were updated according to the data. The model was evaluated on two levels (i.e., the psychosocial health label and the reason for the disorder). After evaluation, it was observed that the model was predicted at an accuracy of 87% with the ability to assign labels depending on the context of the posts. Representative posts and the associated predictions made by the model and the reason for the disorder are shown in Table 7. Additional examples are presented in Supplementary Table S2.

**Table 7:** Sample output of psychosocial health classification.

**Posts**	**Label**	**Reason**
Sorry didn't mean that as an insult. You have been very helpful and kind. I just get very angry at times and my anger kinda takes over	Stress	Getting upset rather easily
My friend's husband has stage IV esophageal cancer. He stopped responding to Chemo. They started immunotherapy. Before Xmas he went to the ER and was admitted a few days. Got a call from ^#^palliativecare today as a follow up. Just like that, she feels less stressed.	Anxiety	Relief when feelings that cause anxiousness end

^#^HumansOfPC.

### 3.4. Symptoms severity

A total of 100 records per symptom for Head & Neck were manually analyzed to evaluate the accuracy of the model in tagging the symptom with posts from Blogs, Forums, Reviews, and Twitter, as well as the sentiments identified with aspects as symptoms from the text. Figure 2A shows the distribution of the symptoms identified by the model across different posts. Then, the aspect-based sentiment model results were analyzed, and human experts defined the symptom severity as the number of posts, where a symptom or impact of a symptom is discussed in negative tonality by the patient or caregiver. Figure 2B shows the severity of such symptoms and their impact on estimating or mitigating the risks as part of the drug development lifecycle.

### 3.5. Potential side effects

Based on the extractions and predictions performed by the model, 250 data points were manually reviewed and analyzed by SMEs to perform qualitative reviews apart from the model-generated accuracy metrics, which can be found here (John Snow Labs., 2021). Post-review and analysis are some of the accurate predictions highlighted by SMEs. Table 8 shows the extraction of the drug and potential side effect value rather than simply labeling whether the text contains potential side effects or not.

**Table 8:** Sample output of potential side effects classification^a^.

**Posts**	**Drug**	**Potential side effect**
Unfortunately I will be going through this with you guys too. I was diagnosed with HPV tonsil cancer of left side with 4 cm clavical lymph. A total of 7 weeks radiation and 6–7 weeks chemo is the plan to start on 11/11 at 11:00 oddly enough. Wild man… I would read up on the 3 dose chemo… I have read higher occurr of permanent side effects such as hearing loss have occurred with that dosing. My Oncologist agreed.	Chemo	Hearing loss
I have Osteoarthritis at c-23,34,45, taking Product A for 2 weeks, gained 16 lbs, Dizziness, lower back pain Hands hot, burning don t know if I have fever are not, chest pain, Incoordination, abnormal thinking, I want to stop taking Product A, because of the wt gain, throat cancer survivor, 1 year, taking Product B 10–325, and morphine 30 mg 2 times a day, I want to ease off Product A, which is 300 mg 4 times a day U will prob say call my dr. mon,	Product A	Dizziness Chest pain Weight gain Abnormal thinking Hands hot Burning Lower back pain
Oh Yea, Product A is well known for causing tinnitus and hearing loss. I was diagnosed with base of tongue cancer in 2011 and went through 3 rounds of “induction” chemo using a cocktail of 3 drugs, one of which was Product C. I already had some ringing from everyday life, but within the year it got pretty noisy. Between my hearing loss and the ringing I went to hearing aids. They help with the ringing as well as allow me to hear better. Most hearing aids have a function to mask ringing if it really bothers you. Fortunately, I seem to tolerate the “crickets” in my ears pretty well. They sing me to sleep every night.	Product C	Tinnitus Hearing loss

^a^Product names have been deidentified.

## 4. Lessons learned and future directions

To summarize, this article highlights the potential benefits of leveraging AI and NLP techniques tailored to PFDD-specific business questions for generating insights into patient experiences at scale. Our article focuses on the development of an AI solution, which is highly customizable and scalable to cater to new indications or therapeutic areas of interest within our organization. This unique solution has a major component for classifying posts into patients and caregivers based on the context of the post, specifically around pre- or post-diagnosis conditions, effects, and experiences with Head & Neck or esophageal cancer. This ML model is retrained based on the concepts of negative learning and is validated by our SMEs. The model was benchmarked at 40% noise level with an accuracy of 87%. Such filtering of posts helps the downstream AI solution to extract insights from qualitative data, thus helping business experts understand patient unmet needs, specific patient populations as target groups, and evidence-generation strategies.

As noted in the literature review, the majority of the research studies in the area of patient experience or PFDD were conducted using sample data or a set of patient interviews with less focus on creating an enterprise-ready, scalable solution for AI-based insights into PFDD. Additionally, our AI solution is evaluated against different geographies and keywords for indication.

The next steps for this solution are to augment and enhance the scope of the data from social media to sources, including, but not limited to, scientific, peer-reviewed publications identified via a literature search strategy, patient interviews, advisory boards, and qualitative and quantitative studies. This way, AI algorithms will be fine-tuned to handle a variety of free texts, resulting in richer recommendations and actionable insights. Furthermore, the models will be validated to understand how quickly they can adapt to newer indications and therapeutic areas of interest. Potential explorations in the areas of large language models and negative learning coupled with biomedical domain-specific embeddings will further enrich the learning from these data sources. Based on the evolution of patient needs and expectations, the models will be fine-tuned to generate AI-enabled decisions that will significantly assist research and development teams to optimize the drug development pipeline effectively and efficiently, making it more patient-driven than organization-driven. As a scale-up to additional therapeutic areas, the solution will be adapted to priority indications of our organization's drug development pipeline.

## Data availability statement

The data analyzed in this study is subject to the following licenses/restrictions: proprietary data. Requests to access these datasets should be directed to prathamesh.karmalkar@merckgroup.com.

## Ethics statement

Ethical approval was not required for the study involving human data in accordance with the local legislation and institutional requirements. The social media data was accessed and analyzed in accordance with the platforms' terms of use and all relevant institutional/national regulations.

## Author contributions

PK contributed to this report in conceptualization, methodology, software, validation, and formal analysis. HG contributed to conceptualization, methodology, and supervision. ES and JF contributed to conceptualization and validation. All authors contributed to writing, reviewing, and editing the manuscript and approved the final version.

## Footnotes

^1^Mayo Clinic. *Mental Illness*. Available online at: https://www.mayoclinic.org/diseases-conditions/mental-illness/symptoms-causes/syc-20374968 (accessed April 12, 2023).
