# Supplementary material for: Artificial intelligence-driven approach for patient-focused drug development
Source: Front Artif Intell. 2023 Oct 12;6:1237124. doi: 10.3389/frai.2023.1237124 (PMC10601646; doi:10.3389/frai.2023.1237124)
Supplement: Supplementary file 1 [file Table_1.pdf]

Supplementary Material

Table S1: Additional Outputs of Medical Sentiment Classification[a]

Posts	Label
I too had a neck dissection whereby a cancerous node and 23 others were removed. Stage IVA (old staging) tonsil cancer with spread into tongue and soft palate and nearby node. Neck dissection and surgery to remove both tonsils and parts of tongue and soft palate was completed 4/20/2016. 33 rads and 7 Product A treatments. Still numb and sore on the neck dissection side after 3 years. Have accepted this may be the case forever at this point. As long as I remain NED, I can live with the numbness and other post treatment issues (thick saliva, etc.)<br>Hope you have many years of NED in your future.	Still exists
I was diagnosed with throat cancer in late November of 2012. Went through treatment starting in 2013 so it is about 7 years. Also had 2 cancer episodes since then a small spot on my tongue removed with clear margins and lymph node cancer last year. Celebrating with the ones that are still here and mourning the ones with their families who have passed-Take Care-God Bless all-	Still exists
Hi Last full week of radiotherapy starts tomorrow and last scan tomorrow which I don't like as your strapped down for longer. Skin on neck and chops starting to get dry and flakey but the barrier cream I was given seems to be moisturising it nicely. Mouth ulcers more or less gone the gel has done wonders especially on my dry mouth. Stopped using mouthwash instead using good old fashioned salt water. Keeping my mouth much cleaner especially my tongue. Cheerio	Still exists
I was blessed for almost 70 years with teeth that rarely needed anything. I had, IIRC 4 fillings and I broke one and ahd to get a crown. Insurance just didn't pay off for me as my only dental expense, really, were the cleanings twice a year.<br>Fast forward to Spring, 2015 and my diagnosis with head and neck cancer, so chemo and 35 radiation treatments. Again, I was blessed that apparently it is gone BUT it did a number on my teeth and my dentist ells me that's not only normal, but will continue for he remainder of my life.<br>Now I have a couple of cavities a year, have had three crowns and get the cleanings every two months (that two months is my idea, just to try to stay ahead of the stuff).<br>I have one tooth the dentist says she cannot save and it's just a matter of time till it's gotta come out.<br>Problem is-I love my dentist and hygienist. They take insurance, but the patient pays the rest. So, if they charge $200 for some procedure and insurance pays $75 bucks, the patient owes another $125.<br>On top of the cost of the procedures, the insurance and its premiums don't make sense for me.<br>I could find another dentist who accepts whatever the insurance company pays plus a deductible, but my dentist is sweet, pretty and, most importantly, pain-free. However, like most really nice things, she's a bit expensive.	Recovery

[a]Product names have been deidentified.

Table S2: Additional Outputs of Psychosocial Health Classification

Posts	Label	Reason
Well … opinions certainly do vary on that subject but some ways are definitely better than others. Last year a friend of mine died from a stroke and it was an incredibly quick death. He didn't suffer, didn't spend a long time worrying about what would happen to his family, didn't deplete all of his family's resource fighting a loosing battle against cancer. It was just lights out one morning. It sucks massively for his wife and 3 kids but it could have been worse. Being on this Earth One Moment and instantly separated from everyone I know and care about here is about as terrifying a thought is I can imagine. As painful of a conditioned esophageal cancer would be at least I would have an opportunity to make some plans and say goodbye to the ones I love as well as having an opportunity to exercise my second amendment rights to make my demise as quick and painless as possible.<br>Life insurance doesn't pay out in the event of a suicide. Ain't that a bitch?	Suicide	Suicide
It must be a worry but I'm honestly not going to say don't worry as it's natural , I myself just became the opposite I went out and socialised. , spent time with my family , deep down like you I had that gut feeling , I'm now 5 months on from sugery for partial tongue removal and neck dissection , won't lie has been a long recovery , but I'm a live cancer free , my speech good , apart from stiffness in neck and shoulder as you can get post op probs when nerves getvdussruopted I'm goid , going back to work soon and very happy and I'm just saying everyday I'm going to live my life now , I do hope all goes ok and you are in my thoughts lots of love my friend xx	Depression	Feeling like you have nothing to look forward to
Throat cancer and neck fusion. Your throat is about the size of a quarter mine is the size of a nickel. Mask can create anxiety when you can't get enough oxygen. I'm not wearing a mask!	Panic	An intense worry about when the next panic attack will happen

Table S3: Sample search queries used to fetch data from SML platform.

Indication	Query
Esophageal Cancer	(“Oesophageal cancer" OR "esophageal cancer" OR "Esophageal adenocarcinoma" OR "Osophageal adenocarcinoma" OR "Adenocarcinoma of the esophagus" OR "Adenocarcinoma of the osophagus" OR "Esophageal squamous cell carcinoma" OR "Osophageal squamous cell carcinoma" OR "Squamous cell carcinoma of the esophagus" OR "Squamous cell carcinoma of the osophagus" OR "Cancer of the foodpipe" OR "cancer of the gullet")NOT("Stomach cancer" OR "gastric cancer")
Head and Neck Cancer	("Head and neck cancer" OR "squamous cell carcinoma of the head and neck" OR SCCHN OR HNSCC OR "Oral Cavity cancer" OR "cancer of the oral cavity" OR "lip cancer" OR "cancer of the upper lip" OR "cancer of the lower lip" OR "larynx cancer" OR "laryngeal carcinoma" OR "Cancer of the glottic larynx" OR "glottic cancer" OR "Cancer of the Supraglottic larynx" OR "supraglottic cancer" OR "Subglottic cancer" OR "pharyngeal carcinoma" OR "cancer of the pharynx" OR "oropharynx cancer" OR "oropharyngeal carcinoma" OR "hypopharynx cancer" OR "hypopharyngeal carcinoma" OR laryngopharynx OR "Salivary gland cancer" OR "Paranasal tumours" OR "Ethmoid sinus cancers" OR "Maxillary sinus cancers" OR "Cancer of the mouth" OR "Cancer of the throat" OR "mouth cancer" OR "gum cancer" OR "tongue cancer" OR "Tonsil cancer" OR "Throat cancer" OR "cancer in the voice box" OR "voice box cancer" OR "Cancer in the vocal cords" OR "glottic cancer")NOT("Nasopharynx cancer" OR "nasopharyngeal carcinoma")

Table S4: Example of cases where algorithm was unsuccessful in identifying correct information from text[a]

Post	Model Name	Failure Reason
We were told without radio 25% chance of recurrence 15% recurrence after radio. The doctor said Generally it would recur locally. I feel at the moment I haven't recovered enough as had an17hr op then got ventilator pneumonia after two days then on day five had to have flap removed and reconstruction from chest another six hr op followed by seven days in ICU. Just feeling I need to get stronger before radiotherapy so think I am going to wait to see if it does recur then take it then. It's just a big dilemma what to do for the best really	Medical Sentiment Classification	Future worries/concerns - patient recently treated and concerned about possible recurrence
Hi there, Totally new to all this, my investigation started just one month ago, now I've finally been informed my cancer is in a lymph node in the neck as well as the gullet so operation is not an option and the cancer is not treatable by conventional Chemo, coupled with an enlarged Thyroid Gland (reason unknown) so the only option for me is palliative treatment with an estimated life expectancy of 3 years, i am 63 now and was looking forward to retirement and spending lots of time with the Grandchildren, this is such a shock to the system that the only way i know to deal with it is staying positive and not denying it exactly but not thinking to much about it. I am lucky as i have some really good colleagues who are always making jokes about the situation which bouys up my spirit no end. Reading some of the comments on here gives me some hope as the palliative Chemo could possibly change my situation for the better and lead to surgery and recovery which i would gladly accept so all the best every body, keep your chin up as best you can, laughter is a good medicine !	Medical Sentiment Classification	Talking about initial diagnosis and life expectancy, not indication of deteriorating in post
I was diagnosed with oesophageal cancer (at distal end/stomach entry) in May, 2018. Chemo-- 9 sessions of Drug S and Drug T) and radiation (28 sessions on Drug V)- - chemo and radiation all completed over 6 week period. I was able to swallow again after about 2-3 weeks. Now more than 2 years later, I have no cancer based on CT scans.	Psychosocial Health Prediction	Mentioned symptoms are all side-effects to treatment, no mental illness

I do have some continuing after effects-- all tolerable...and I am very grateful... every day is a good day.<br>I do have fatigue-- difficult to explain...but if I force my self to ""get going and do something"".. the fatigue is put in the background...but each morning the fatigue is back again...and I have to get over it again.<br>Shortness of breath was quite severe for several weeks-- caused by scar tissue radiation damage to both bottoom lobes of the lung. Much improved now.<br>Dry mouth-- each night I wake up due to dry mouth-- sugar-free cough drops helps.<br>Constipation-- never a problem during treatment and afterwards, but I have been in the habit of adding one tablespoon of dextrin fiber to my bran cereal each morning for years.<br>Aches and pains-- tolerable without any medication. How much is cancer treatment side effects or age-- ? I am 86 years old.<br>Blood tests-- I continue to be just below normal for R, W, hemoglobin, and lymphocytes-- so immone systems is compromised.<br><br>I hope you do well-- as good as my condition, or better!		
You are 1000% not alone. Just in the last week I've "had" skin cancer, lymphoma, leukemia, and throat cancer. When I go to the doctor and he "rules out" my fear I either don't believe him or jump to a new one shortly after. I got prescribed Drug P today.	Psychosocial Health Prediction	Likely anxiety instead, feelings of fear surrounding getting cancer
Hi gEm ! I have recently been diagnosed and am awaiting a PET and second endoscopy. I too have had greatly reduced appetite, feeling full etc. Eating more often with smaller portions, making things slippery with gravy, ketchup etc. and using Ensure occasionally all are helping me to maintain my weight. My Dr. Has said it is important to maintain weight before treatments start. Ice cream and frozen yogurt bars are great too!!! All the best to you and your Dad!	Potential Side Effect	Patient did not undergo treatment yet; Describes symptoms/impacts related to disease

[a]Product names have been deidentified.
